# Retrospective evaluation of 377 patients with penetrating foreign body injuries: a university hospital experience (a present case of missed sponge foreign body injury)

**DOI:** 10.3906/sag-2006-34

**Published:** 2021-04-30

**Authors:** Anıl Murat ÖZTÜRK, Omar ALJASIM, Gamze ŞANLIDAĞ, Meltem TAŞBAKAN

**Affiliations:** 1 Department of Orthopedics, Faculty of Medicine, Ege University, İzmir Turkey; 2 Department of Infectious Diseases and Clinical Microbiology, Faculty of Medicine, Ege University, İzmir Turkey

**Keywords:** Foreign body, sponge, missed foreign body, complication, injury

## Abstract

**Background/aim:**

This study aimed to retrospectively analyse patients with foreign body (FB) injuries in our hospital and to present a patient with missed penetrating sponge FB injury.

**Materials and methods:**

This study lasted 12 years (2008–2020) and reviewed all patients with FB injuries who were admitted to the emergency department (ED) of our hospital. Along with our overall results, we present a case with missed penetrating sponge FB injury in detail.

**Results:**

Approximately 377 patients were included in the study (age: 28.3 ± 18.3 years, m/f: 229/148). The foot (n = 148, 39.3%) and the hand (n = 143, 37.9%) were the most frequently injured body parts. Regarding FB types, sewing needles (n = 140, 37.1%), metal pieces (n = 91, 24.1%), and glass (n = 80, 21.2%) were the most frequently observed objects. Most of the patients were injured at home, often by needles or glass. The injury-admission mean time was 7.38 ± 2.5 days. FBs were frequently removed in the ED (n = 176, 46.7%). Plain radiography is the first line in identifying FBs. Soft tissue infection was the most common complication. MRIs were much useful than USGs in detecting the missed penetrating sponge injury of the single patient in the study.

**Conclusion:**

For diagnosis of FBs, besides recording the patient’s history, obtaining a two-sided radiogram is of great importance. For nonradiolucent or deeply located FBs, further clinical or radiological investigation must be considered to avoid complications. Although most of the FBs can be removed in the ED, patients may require hospitalisation and operation for FB removal, depending upon FB location and age.

## 1. Introduction

Foreign bodies (FBs) are an important reason for attendance at emergency departments (ED) [1]. Retention of FBs can cause some complications as inflammation, infection, and damage to surrounding structures. Therefore, removal of FBs is crucial. Early diagnosis and prompt removal of FBs is required to prevent complications. Superficially located FBs can easily be retrieved with wound exploration under sterile conditions by adequate local anesthesia in EDs. In some deeply located FBs, further inspection of the wound and deep dissection with local anesthesia can be challenging for the surgeon [2,3]. Surgery is required in these situations. Radiographs have an important role in localising FBs and are initiated for the initial assessment of radiopaque FBs for type and location. However, nonradiopaque FBs like wood or plastic are not visible with normal radiographs [4,5]. Furthermore, the size of the FB can be so small that it cannot be identified with a standard x-ray examination. It is crucial to know that the exact location of the FB in particular proximity to tendons, neurovascular structures, and other visceral structures in order to make surgical dissection effective and to not damage healthy adjacent structures. A badly planned or poorly carried out dissection can cause redundant hazard of soft tissue, elevate the risk for infection, and impair wound healing. Furthermore, undetected FBs will lead to worse patient outcomes, increased inpatient costs, long hospital stays, and repetitive surgery [6]. Even in immunocompromised patients, FB injuries with bacteria seeding can be a cause for necrotizing fasciitis, a serious morbidity risk [7]. Most of the studies related to FBs in the literature are made up of case reports. There is a lack of FB-related research about comorbidities, the microbiological cultures of complicated cases, injury location, second operation rates, and hospital stays. Furthermore, case reports about undetected wooden FBs are not very common. Although there have been studies conducted on retained FBs during surgery—mostly with a laparotomy sponge—to the best of our knowledge, there has not yet been a published paper about undetected penetrating sponge FB injuries in the literature. This study presents a patient with a missed sponge FB in a lower extremity, something that has not previously been found in the literature. This study aimed to retrospectively analyse patients with FB injuries and also present data about a patient with a missed penetrating sponge FB injury.

## 2. Materials and methods

**Figure 1:**
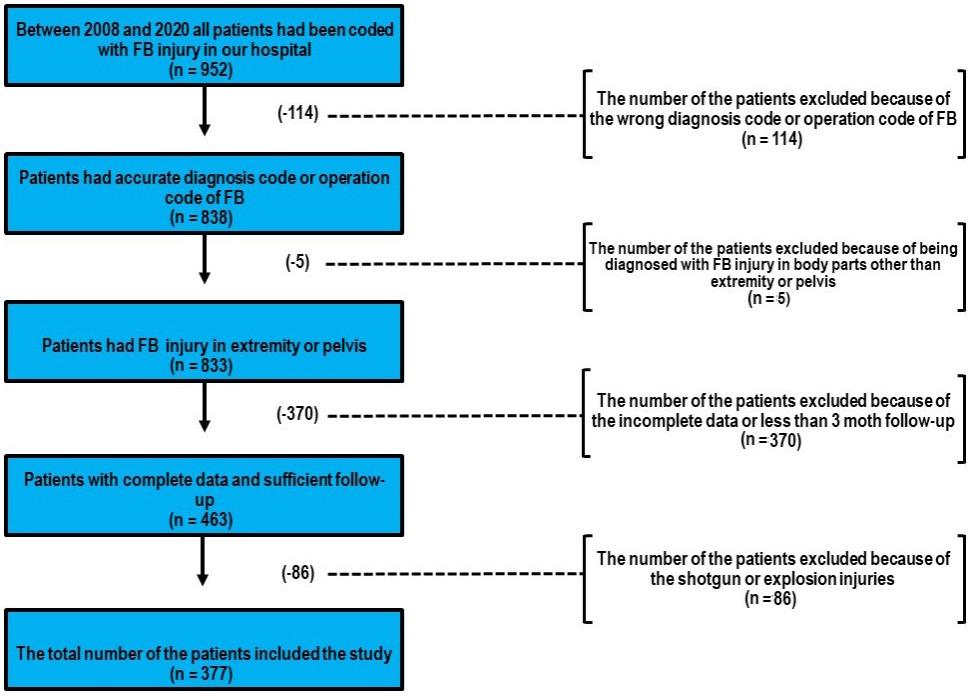
Patients excluded from the study and reasons for their exclusion. Although we had 952 patients with FB-associated problems, after the evaluation of patient files 575 patients were excluded and eventually the total number of patients included in the study was 377.

This study reviewed all patients with FB injuries who were admitted to the ED of our hospital between January 2008 and January 2020. The International Classification of Diseases Specific Codes manual (ICD-952, World Health Organization, Geneva, Switzerland) was used to estimate patient accrual rates. The inclusion criteria were patients with an FB injury of the extremity or pelvis and who had sufficient data in their electronic archive. Patients with insufficient data in their electronic archive or with high-impact injuries such as bullet injuries were excluded from the study. A total of 377 cases (229 men, 148 women) that met the inclusion criteria were enrolled in the study (Figure 1). Demographic and clinical data such as age, sex, injury location, comorbidities, type of FBs, the place where the injury occurred, the department where the FB was removed, radiological investigations, presence of abscess or osteomyelitis, anesthesia types, antibiotic usage types, tetanus vaccine application, the time that elapsed between the incident of trauma and intervention, other clinic applications, interventions before the patient applied to our centre, type of complication, number and reason for reoperation, and length of the hospital stay were all recorded. 

Regarding the radiological evaluations of the patients, the depth of the FB was recorded as ‘deep’ if it was near vital structures, beneath the fascia, near bone, or inside the muscles; otherwise, it was recorded as superficial. Operations for all removals of FBs were made by orthopedic surgeons. The setting of the operation and anaesthesia type were grouped as follows: ED, operating theatre or local anesthesia, regional nerve block, sedation, spinal anesthesia, and general anesthesia, respectively. Along with our overall results, we present a case with a missed penetrating sponge FB injury in detail. 

### 2.1. Statistical analysis

All analysis was done in Excel and SPSS 23. Percentages and means ± standard deviation were calculated to describe the distributions of categorical and continuous variables, respectively.

## 3. Results

Among the 377 patients, who were between the ages of 1 to 83 (28.3 ± 18.3) years old, male patients (n = 229, 60.7%) were more numerous than female patients (n = 148, 39.3%). The injuries occurred mostly on the left side (n = 203, 53.8%). Although most of the patients had no comorbidities (n = 296, 78.5%), hypertension (n = 23, 6.1%) and diabetes mellitus (DM) (n = 28, 7.4%) were frequently observed comorbidities in the study (Table 1). 

**Table 1:** Demographic data, injury side, comorbidities, FB types, injury location, where the FB was removed, injury-diagnosis time analysis, and hospital stay.

		Number	%
Side	Right	174	46.2%
	Left	203	53.8%
Comorbidity	No comorbidity	296	78.5%
	Hypertension	23	6.1%
	Diabetes mellitus	28	7.4%
	Atopic dermatitis	3	0.8%
	Pulmonary diseases	13	3.4%
	Hematological disorders	2	0.5%
	Obesity	1	0.3%
	Rheumatologic diseases	1	0.3%
	Neurological diseases	7	1.9%
	Endocrine disorders	1	0.3%
	Malnutrition	1	0.3%
	Live diseases	1	0.3%
Object type	Needle	140	37.1%
	Glass	80	21.2%
	Metal	91	24.1%
	Nail	13	3.4%
	Wood	23	6.1%
	Crochet hook	9	2.4%
	Plastic	3	0.8%
	Cement	1	0.3 %
	Fishhook	5	1.3%
	Bone marrow needle	2	0.5%
	Sponge	1	0.3%
	Knife	5	1.3%
	Screwdriver	1	0.3%
	Drill bit	1	0.3%
	Metal balustrade	2	0.5%
Where the injury occurred	Home	250	66.3%
	Outdoor	45	11.9%
	Work place	79	21%
	Hospital	2	0.5%
	School	1	0.3%
Where the FB removed	Emergency department	176	46.7%
	Operating theatre	160	424%
	Did not removed	39	10.3%
	Removed in another department	2	0.5%
Hospital stay (day)	minimum	maximum	mean
	2	138	11.47
Injury - admission time	< 24 h	> 24 h < 1 weeks	> 1 week
	308 patients	40 patients	29 patients

**Figure 2:**
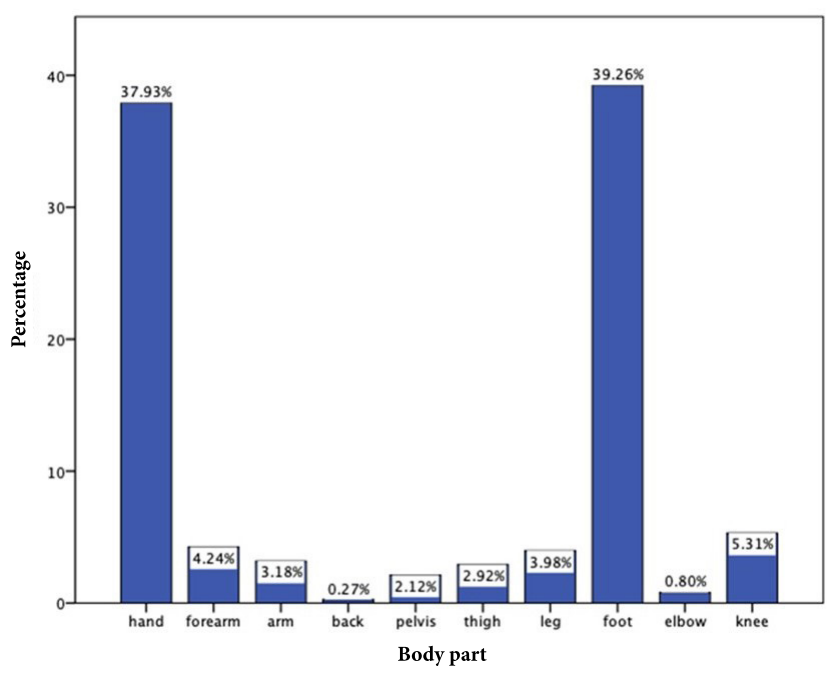
Distribution of FBs depending on the site/location of injury.

**Figure 3:**
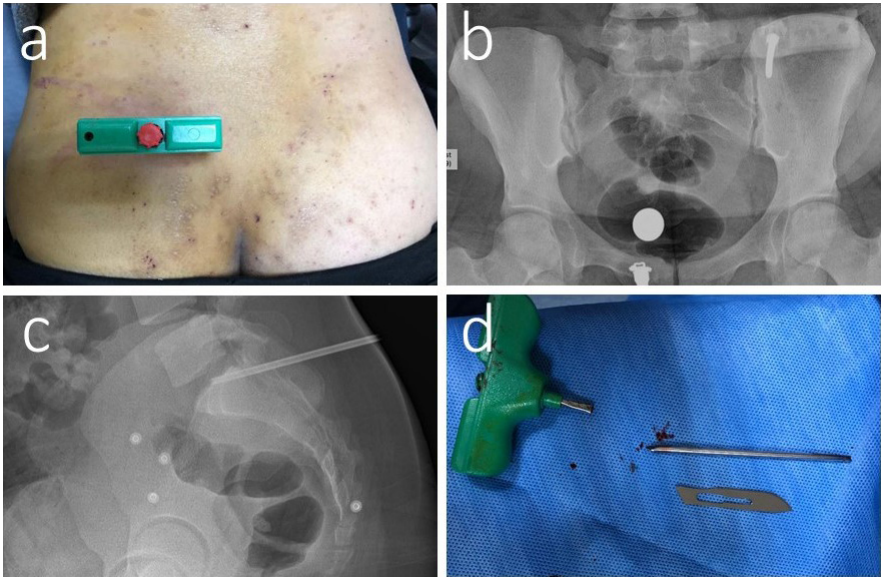
29-year-old female patient with a diagnosis of aplastic anemia. A bone marrow biopsy was performed on the patient (a); during the biopsy the needle was broken, and we removed the FB (b,c); under local anesthesia, the broken needle was removed (d).

The foot (n = 148, 39.3%) and the hand (n = 143, 37.9%) were the most frequently injured body parts (Figure 2). In terms of FB types, sewing needles (n = 140, 37.1%), metal pieces (n = 91, 24.1%), and glass (n = 80, 21.2%) were the most frequently observed objects. Most of the patients were injured in their homes (n = 250, 66.3%), mostly by needles or glass. Injury in the workplace (n = 79, 21%) was the 2nd most frequent place of injury, and FBs in these cases were mostly metal pieces. Street injuries (n = 45, 11.9%) were mostly caused by glass or wood. Two injuries occurred at the hospital during bone marrow aspiration, and the needle was removed by an orthopedic surgeon (Figure 3, a–d). One patient had a metal body injury that occurred in a school workshop (Table 1).

FBs were frequently removed in the ED (n = 176, 46.7%). When removal in the ED failed, FBs or deep FBs were removed in the operation theatre (n = 160, 42.4%). Some patients (n = 39, 10.3%) refused the operation, and these patients were given oral antibiotics. The majority of the patients (n = 308, 81.7%) were admitted to the hospital <24 h after injury. Approximately 40 (10.6%) patients were admitted during the 1st week, while 29 (7.7%) patients were admitted after 1 week following injury occurrence. Injury-admission mean time was 7.38 ± 2.5 days. Sixty (15.9%) patients were admitted to other centres prior to admission to our hospital and some had developed complications with FB injuries. Two patients developed abscesses after FB removal, and one patient was admitted with soft tissue infection. Approximately 137 patients had outpatient surgery. Twenty seven (7.2%) patients needed hospitalisation because of IV antibiotic therapy. The duration range of hospital stay for these patients was from 2 to 138 days (mean: 11.47 ± 26.76 days) (Table 1). 

**Figure 4:**
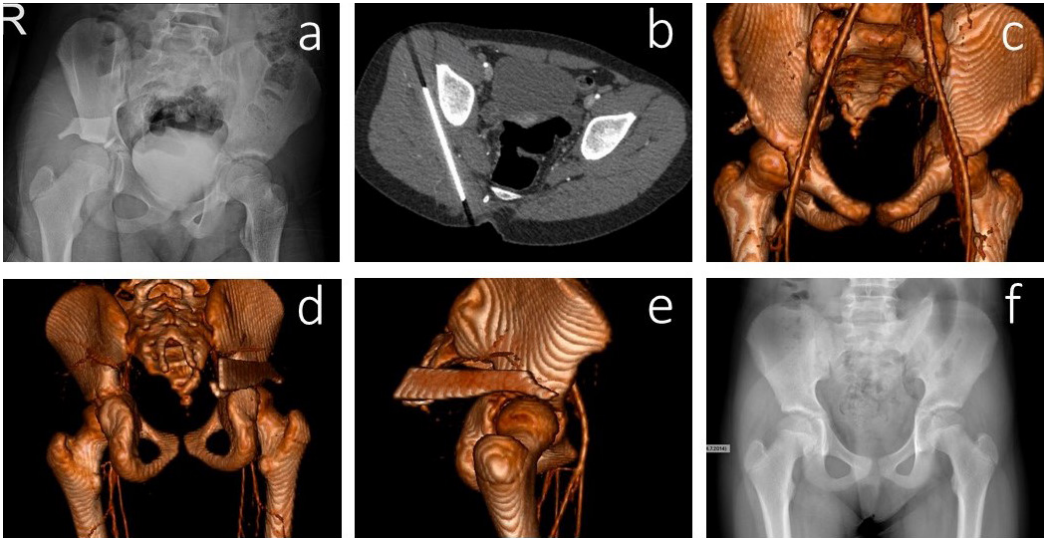
15-year-old female patient with a glass injury on her right hip. Although the FB was clearly visible on the radiogram (a), we initiated a CT of the patient in order to decide on the exact anatomical location of the FB (b); moreover, because of the complex anatomy of the region and morphological variations, an angiography was done to exclude any vital organ injuries, look at the relation to the hip joint, and to plan for her operation (c–e). Under general anesthesia, the glass pieces were removed without any neurovascular injury. Postoperative radiography of the patient (f).

**Figure 5:**
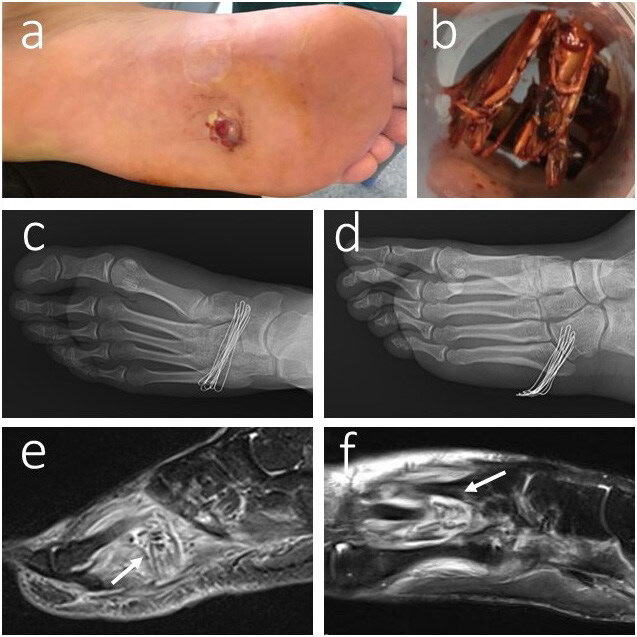
20-year-old male with a wood injury on his left foot. He was admitted 1 week earlier to another hospital and discharged with oral antibiotic therapy only. On his admission to our hospital, there was a discharge, redness, and swelling on his left foot (a); the radiographic image showed nothing related to the FB (c,d); C-reactive protein was 5.23 Mg/L, leucocyte 11.13 × 10^3/μL, and the erythrocyte sedimentation rate were 16 mm/h. Because of this, we decided to get an MRI of the patient, and the MRI-T2 showed abscess formation around the FB (axial view (e) and sagittal view (f)); the wood pieces (diameter range: 4 mm–70 mm) were removed from his left foot (b).

**Figure 6:**
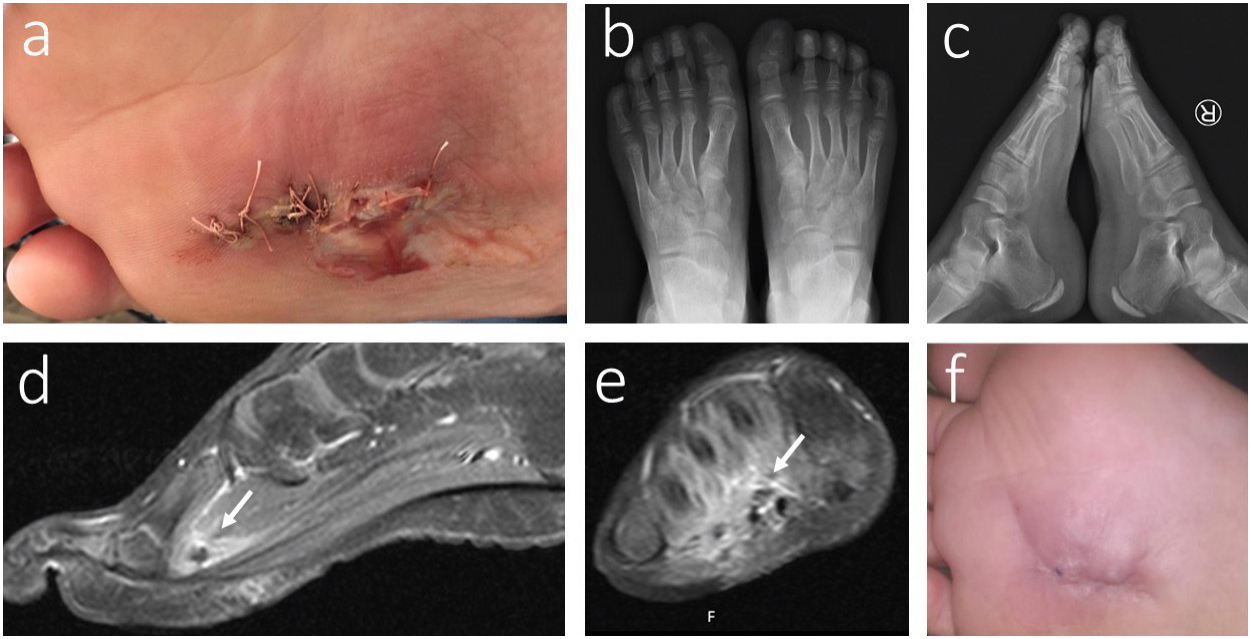
11-year-old male with missed penetrating sponge injury in his right foot. On his admission to our hospital, there was a purulent discharge on his right foot after his first administration to another centre (a); radiographic images showed nothing related with the FB (b,c); ultrasonography revealed 2 FBs in his right foot. After removal of the FBs, a purulent discharge continued up to the follow up period. An MRI was performed for the patient, and the MRI-T2 showed a missed FB between the 2nd and 3rd flexor tendons of the foot (sagittal view (d), axial view (e)); therefore, we understood that the USG did not locate the 3rd FB, and the patient was operated on to remove the 3rd missed sponge FB. The wound healed, and the discharge stopped after the removal of the deep missed FB after the 2nd operation in our hospital.

The location of FBs was often determined by the mechanism of the injury. Radiolucent FBs like wood, glass, and plastic were localised based on patient history and findings from the physical examination. When the penetrating object was nonpalpable or could not be observed superficially, patients underwent radiologic investigation. First, according to the protocol, we performed a direct radiography of the patients. If the FB was radiolucent or if there was a risk of additional complications concerning FBs such as infection, localised cellulitis, and abscess formation or whether the FB was near a vital structure, then other radiological investigations were made such as a USG, CT, MRI, or CT angiography. In most patients, the FBs were detected with normal radiography (n = 353, 93.6%). For radiolucent objects, underlying pathologies, or suspicion of vital organ injuries, advanced radiological investigations were used. In a 15-year-old female patient with a glass injury on her right hip, although the FB was clearly visible on the radiogram, we initiated a CT scan of the patient in order to decide the exact anatomical location of the FB. Moreover, because of the complex anatomy of the region and morphological variations, an angiography was done to exclude any vital organ injuries related to the hip joint and to plan for her future operation (Figure 4a–4f). In 2 patients, FBs were detected by MRI before surgery (Figures 5a–5f and 6a–6f). 

FB injuries were superficial in 225 (59.7%) patients, while 134 (35.5%) patients had deep injuries. Usually, most superficial FBs were removed under local anesthesia in the ED or in an outpatient clinic. If the FB failed to be removed or any deep FB was suspected, the patient was scheduled for operation. FB removal was mostly performed under fluoroscopic control. Accordingly, the FBs observed at the EDs and several in the operation theatre were frequently removed under local anesthesia (n = 201, 59.5%). The remaining cases were removed in the operation theatre under regional nerve block (n = 57, 15.1%), sedation (n = 43, 11.4%), general anesthesia (n = 20, 5.3%), and spinal anaesthesia (n = 17, 4.5%) (Table 2).

**Table 2:** Radiological tests, depth, and anesthesia types. Evaluation of the microbiology culture, antibiotic usage, and tetanus vaccination.

		Number	%
Radiological test	X-Ray	353	93.6%
	No radiological test	14	3.7%
	X-Ray and CT	4	1.1%
	X-Ray and MRI	3	0.8%
	X-Ray and CT angiography	2	0.5%
	X-Ray, USG and MRI	1	0.3
Deepth	Superficial	225	59.7%
	Deep	134	35.5%
Anesthesia type	Local anesthesia	201	59.5%
	Regional nerve block	57	16.9%
	Sedation	43	12.7%
	General anesthesia	20	5.9%
	Spinal anesthesia	17	5%
Culture	No culture taken	360	95.5%
	Culture positive	8	2.1%
	Culture negative	9	2.4%
Antibiotic usage	Antibiotic used	112	29.7%
	No antibiotic used	265	70.3%
Tetanus vaccination	Vaccinated	109	28.9%
	Nonvaccinated	268	71.1%

**Figure 7:**
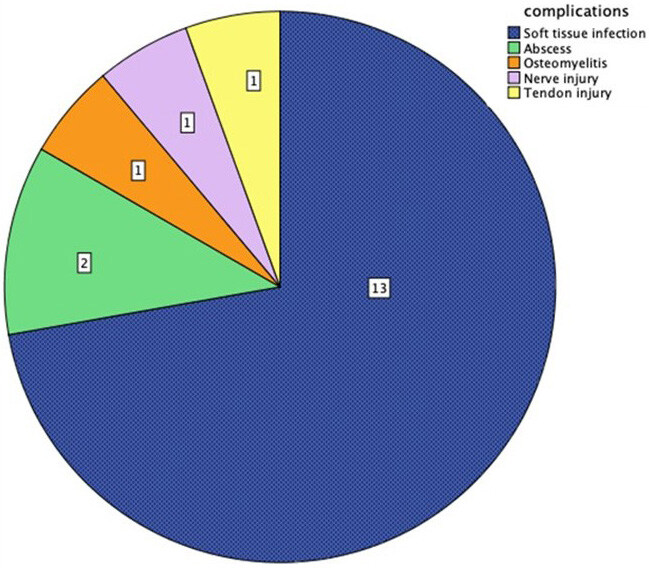
Complications of FBs frequencies.

**Figure 8:**
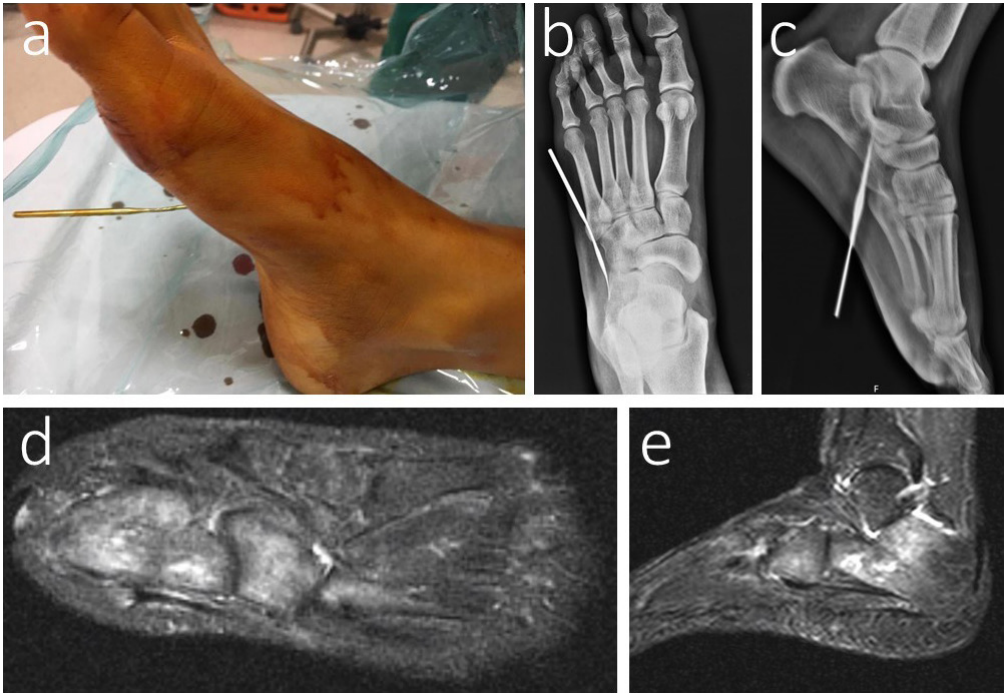
52-year-old female patient with crochet hook injury in her right foot (a); radiographies showed the direction of the crochet hook in her right foot (anteroposterior view (a), lateral view (b)); after removal of the FB, the patient was discharged with oral antibiotics. She had DM as a comorbidity. At 2 weeks follow-up, the MRI-T2 axial view showed osteomyelitis of calcaneus, cuboid, and 5th metatarsal bones (d,e).

Complications were observed in 18 (4.8%) patients (Figure 7). Soft tissue infection was the most common complication and was detected in 13 (3.4%) patients. Two patients were diagnosed with an abscess. One of them was a missed wood injury treated surgically (Figure 5 a–f). One patient had median nerve neuropathy after a glass injury, and another patient had extensor tendon laceration after a glass injury. One patient developed osteomyelitis of the right foot after a crochet hook injury. This diagnosis was confirmed by MRI (Figure 8a–8e). 

From 17 microbiological cultures, 8 had positive results. *Staphylococcus aureus* was the most common pathogen isolated. Antibiotics were prescribed for 112 patients. For the rest of the cases, there was no need for antibiotics. According to the guidelines, 109 patients received a tetanus vaccine (Table 2).

### 3.1. Presenting of the patient with missed penetrating sponge FB injury

An 11-year-old male patient was admitted to our hospital with a missed sponge injury of the right foot. According patient’s history, he was previously admitted to another centre and evaluated with a plain radiogram of the affected side. Because the FB was not detected in his biplane radiographs, he was discharged with only oral antibiotic therapy. On his admission to our hospital, there was a purulent discharge on his right foot. The biplane radiographies showed nothing related to the FB. We chose to continue with an USG, and it showed 2 FBs with soft tissue edema, suggesting the presence of infection. Two FBs detected by USG were removed surgically, and IV antibiotic therapy was started. However, during the follow-up period, the purulent discharge continued. Following this, an MRI was needed to rule out other causes of the problem. A T2-weighted MRI showed an FB between the 2nd and the 3rd flexor tendons of the foot surrounded by fluid collection. A 2nd operation was performed and, besides debridement, the missed 3rd sponge FB (3/5/4 mm) was also removed with loop dissection of the plantar aspect of the patient. The wound healed, and the discharge stopped after the removal of the deep missed FB after the 2nd operation in our hospital (Figure 6a–6f). The injury, due to the missed penetrating sponge material, was the cause of the wound infection. The migration of 1 deep sponge piece led to the nonresolving complications. Ultrasonography requires prior training, an understanding of anatomy, and clinical time. Objects may be mistaken for anatomic structures such as tendons, vessels, or bursa, especially in hands, feet, or joints. Objects deeper than 2 cm will also be more difficult to identify as imaging that goes deeper into tissue leads to decreased resolution. The requested ultrasound study indicated negative results, and the diagnosis was made with an MRI.

## 4. Discussion

In this study, we present one of the largest single-centre FB injury series in the literature and also present a patient with a missed sponge FB in a lower extremity, something that was not presented in the literature before. When the literature is examined, most publications related with FB injuries are in the form of case report studies. The authors did not present comorbidities, microbiological cultures of complicated cases, injury location, second operations, or hospital stay. In this report, we present the results of our cohorts, consisting of 377 patients. The clinical manifestations, types of radiology investigations, treatment modalities, and complications were evaluated.

Most of the FBs located superficially can easily be detected with a broad wound exploration and physical exam. However, in some deeply located ones, it is hard to establish and retrieve FBs only with a clinical examination. In these situations, radiological investigations are required to identify the FB and adequately demonstrate the exact location in order to choose the appropriate surgical approach. Conventional radiography remains the first-line investigation for the initial imaging modality because of the success in easily detecting radiopaque FBs in a cost-efficient way with comparatively low doses of radiation. However, the level of visibility of small objects having similar densities as bone or very close to bone can be hard to recognise. Furthermore, the accuracy of radiography in detecting radiolucent FBs like wood or plastic is poor. Plain radiographs in 2 projections are efficacious in detecting all FBs with a success rate of 80% [8]. Nevertheless, the success rate of the plain radiographs in at least in 2 projections of wooden FBs decrease to as low as 14% [9]. In these situations, ultrasonography is a rapid and affordable imaging modality for detection of such radiolucent FBs. Although an MRI is a better alternative to ultrasonography, when wooden FBs are diminutive and there is a lack of associated noninfected fluid collection or abscess, it can be less accurate in terms of identification [10,11]. Furthermore, an MRI is more expensive, less readily available, and is more time consuming. Additionally, an ultrasonography evaluation provides considerable data, including the size and depth of FBs and anatomic relations with adjacent tissues [12–14]. Although the CT is another alternative radiological investigation, with sensitivity 5–15 times greater than that of a plain radiography, it may not be as sensitive and reliable as an ultrasonography or an MRI. Additionally, because of the time needed for scanning, radiation exposure, expense, and less availability, the use of a CT is not widespread in clinical settings [15]. In contrast, some previous studies have indicated that a CT is a better radiological investigation for detecting plastic material bigger than 0.5 mm, followed by a USG and then an MRI [11,16]. In our study, different diagnostic investigations were also used as needed and described in Table 2. Several types of radiolucent FBs such as wood or sponge remain undetected with an evaluation consisting of only conventional radiography. Furthermore, in our study, more advanced radiological investigations were applied to exclude complications or vital organ injuries and to additionally plan for surgery. In cases with deeply-located FBs in the pelvic region, besides a direct radiography, a CT evaluation of the patient’s affected side is important to exclude vital organ injuries and to plan for the surgical approach. An MRI is an essential component in the evaluation of a patient with suspected osteomyelitis or abscesses. (Figures 4a–4f, 5a–5f, 6a–6f, and 8a–8e). 

As we described in the clinical course of the patient whose case involved a missed sponge FB, the MRI was superior to the USG evaluation in detecting radiolucent FBs, especially ones with small diameters, and this is different from the literature. In our patient, the injury leading to a missed penetrating sponge material was the major cause of wound infection. The migration of 1 sponge piece led to the nonresolving complications. The superiority of the MRI over ultrasonography can be explained by the fact that ultrasonography requires prior training as the required skill needed for observation could hardly be achieved with an understanding of anatomy only. With an ultrasonographic evaluation, FBs may appear as tendons, vessels, or bursa, especially in hands, feet, or joints. Besides, objects deeper than 2 cm will also be more difficult to locate because of decreased resolution. This is why in our patient the ultrasound evaluation revealed negative results, and the diagnosis of a missed sponge FB was made with an MRI. The USG failed to detect a deep FB later observed by MRI due to soft tissue infection and fluid collection around a small piece of sponge. A similar case was reported with a different scenario 2 years after injury [17]. Previous comparative studies of different radiological investigations were done in vitro. Comprehensive studies are needed to compare different radiological investigations for FB injuries. 

FB injury was frequently observed in the foot (n = 148; 39.3%) and the hand (n = 143; 37.9%). These results were similar to previous data in the literature [18–20]. In some situations, adjacent tendons can be affected by FBs and irritated or septic tenosynovitis can occur. Even infectious tenosynovitis can result from direct inoculation of an FB. If an FB harms or is close to the nerve, it can cause some complications like neuromas or neuropathies [21]. With the migration risks, FBs have the capacity to move to deeper into the soft tissues of the body such as fascia, ligaments, and joint capsules or even into blood vessels [22–24]. FBs that penetrate near or directly into the bone may cause osteomyelitis with direct inoculation of bacteria [25]. In the current study, complications were observed in 18 (4.8%) patients. Soft tissue infection was the most common complication, and it was detected in 13 (3.4%) patients. Two patients were diagnosed with an abscess. One of them had a missed wood injury treated surgically (Figure 4a–4f). One patient had median nerve neuropathy after a glass injury, and another patient had extensor tendon laceration after a glass injury. Both of these patients’ complications were related with the type and location of the FBs. Only one case, a 52-year-old female patient with a crochet hook injury in her right foot resulted in osteomyelitis. The radiographic image showed the direction of the crochet hook in her right foot. The FB was close to the calcaneus and cuboid. After removal of the FB, the patient was discharged with oral antibiotics. She had diabetes mellitus as a comorbidity. Although early removal and adequate antibiotherapy occurred, at 2 weeks follow-up she developed osteomyelitis related to the penetrated FB (Figure 8a–8e). 

In this retrospective study of 377 patients, injuries frequently occurred in males with a mean age of 28.3 ± 18.3. Timmers et al. described FB injuries in a pediatric population of 8149 cases. In this study, the male population is marginally involved [26]. Potini et al. described hand injuries, predominantly in males with an average age of 38 years [27]. To our knowledge, there is no study in the literature using a high population describing FB injuries related to the extremities in different age groups. 

Time from injury to admission ranged from several hours to 720 days. Usually, injuries with missed FB penetration led to neglected objects. In time, neglected bodies may develop some complications such as infections or can even be detrimental to adjacent structures [28,29]. Those complications necessitate that patients be admitted to a hospital. In our study, a patient diagnosed with median nerve neuropathy was admitted to our hospital 3 weeks after the injury and was scheduled for operation. During the operation, we noticed a reaction caused by the FB that produced a space-occupying mass lesion that was the cause of his symptoms. A history of FB injury was revealed by retrospective direct questioning. Choudhari et al. previously reported a similar case [21].

All of the complicated injuries had deep microbiological cultures taken during surgery. Nine had negative results treated with empirical antibiotics. Operative specimens were taken for cultures. Eight patients had positive culture and were treated depending on the type of organism. In one patient with an extensor tendon laceration, there was no need for culture. *Staphylococcus aureus* was the most common pathogen isolated in the cultures. *Staphylococcus aureus* has been described as a pathogen in some case reports, while another study described pseudomonas as a common organism isolated after foot penetrating injuries [30,31]. Patients with positive culture were treated with antibiotics according to the culture results. With the exception of some case reports and case series, microbiological culture analyses have not been mentioned in any other comprehensive study before. 

Most of the FBs were removed during outpatient surgeries. Twenty-seven patients had a hospital stay with a mean of 11.67. Those patients mostly had to have IV antibiotics or they had other medical conditions. One patient who had a soft tissue defect after a car accident was treated with a free anterolateral thigh flap. This individual had a prolonged hospital stay (138 days), and this affected our results regarding hospital stay. We could not find any data regarding hospital stay described before in the literature. 

Sixty patients were admitted to other hospitals before. Two patients developed an abscess after FB removal and 1 patient had a soft tissue infection. In those patients, we noticed missed FB remnants. Similar cases have been described after incomplete removal of FBs [15,30,32]. A comprehensive history, careful examination, and invasive investigation may be needed to decrease missed FBs. 

Although early diagnosis and prompt removal of FBs are required to prevent complications, there is no consensus about the approach for detecting FBs. Recently, a procedure was reported in the literature about identifying FBs [17,33,34]. Although radiography holds excellent sensitivities for radiopaque FBs, the accuracy, sensitivity, and positive predictive value of ultrasound in detection of nonradiopaque FBs were found to be 94%, 99%, and 94%, respectively [35,36]. Interestingly, a recent paper by Braig et al. demonstrated the success of dark-field radiography for the detection of wooden FBs in a human hand sample. They claimed that this procedure would increase the success of nonradiopaque FB identification by radiography only by easing the efforts in diagnosis [37]. Erik A et al. stated that all wounds harbour the potential for FBs, and if the clinician or the patient has a reasonable level of suspicion, the next step should be to obtain plain film radiographs with views in at least 2 projections. If the exam is negative and only radiopaque objects (gravel, glass, or metal) are suspected, a provider may stop here. However, if radiolucent objects such as thorns, wood, or plastic are suspected, an ultrasound examination of the area should be performed. If the FB is still not located, the clinician may choose to move on to a CT or an MRI, depending on the level of suspicion or type of FB [38]. Furthermore, in recent studies, ultrasound imaging or application of navigation and positioning systems were used intraoperatively to manage the approach in improving the localisation of radiolucent FBs with a high accuracy [39,40]. According to our management, the location of FBs was often determined by the mechanism of the injury. When the penetrating object was nonpalpable or could not be observed superficially, patients underwent radiologic investigation. According to the protocol, we first performed two-plane direct radiography on the patients. If the FB was radiolucent or if there was a risk of additional complications concerning FBs such as infection, localised cellulitis, or abscess formation or in order to establish whether a FB object was near vital structure, other radiological investigations were made such as USG, CT, MRI, or CT angiography. In 14 patients (3.7%), the FBs were detected by physical examination only. However, in most patients (n = 353, 93.6%), the FBs were detected with plain radiography. For radiolucent objects, underlying pathologies or suspicion of vital organ injuries, advanced radiological investigations were used. We preferred USG only in 1 patient with a missed sponge FB injury, but it failed to detect the FB. In this case, the FB was detected with an MRI. In 4 patients, although the FB was clearly observed in the radiogram, we decided to get a CT of the patient in order to decide on the exact anatomical location of the FB and to plan the surgical approach. Moreover, in 2 patients, because of the complex anatomy of the region and morphological variations, an angiography was done to exclude any vital organ injuries and to plan for operation. Besides radiography, in 3 patients, MRIs were useful to diagnose FB-related complications such as abscesses and osteomyelitis. Moreover, as we presented in the single sponge-related incident, an MRI was superior in detecting a sponge FB. In our clinic, we are not able to use an intraoperative ultrasound imaging or navigation and positioning system to detect FBs during surgery.

There has only been one study conducted about the different types of penetrating FBs injuries. Tuhan et al. reported that FBs removed from extremities included 216 needles, 33 metal pieces, 28 glass pieces, 10 wooden pieces, 4 plastic pieces, and 4 stones [20]. Similarly, in our study, sewing needles (n = 140, 37.1%), metal pieces (n = 91, 24.1%), and glass pieces (n = 80, 21.2%) were frequently observed objects. There were no patients with stone FB injuries in our study.

Several limitations of this study should be acknowledged. First, outcomes could have been influenced by recall bias. Since our study has a retrospective design, prospective randomised trials are needed to evaluate the complications and establish a guideline to remove FBs successfully. 

## 5. Conclusion

No procedure is unique in detecting FBs. Because of this, it is important to do an extensive physical examination of the wound and also to choose a convenient, proper radiological investigation. Early diagnosis and appropriate management of missed FBs can be of great help in preventing serious consequences. When swelling or discharge following the removal of FB has been diagnosed, it should be considered that this could be a recurrent infection related with a retained FB. For nonradiolucent FBs, advanced radiological investigations must be taken into account to prevent complications. Although most FBs can be removed in the ED, depending upon the site of FB and age of the patient, hospitalisation and operation for FB removal may be required later. To prevent complications related to missed or retained FBs, further algorithms are needed for the diagnosis of FBs. 

## Ethics approval

Ethics approval for this study was obtained from our university’s Human Research Ethics Committee (20-2.1 T/5).
